# The Moral Condition of London

**Published:** 1900-02-10

**Authors:** 


### THE MORAL CONDITION OF LONDON.

A crowded and influential public meeting, convened by a newly-formed body known as the London Council for the Promotion of Morality, was held last week to consider the moral condition of London—truly a large and almost inexhaustible subject. The chair was taken by the Bishop of London, who said that their first desire was to cope with organised temptations to immorality. At present, he said, things were certainly bad, as no one could deny who took a walk through the streets in the evening, but he thought that this was largely due to the fact of secret haunts of vice having been closed, and that, at any rate, we were now tolerably sure to see the worst of things.

It is to be feared that we have all become hardened by use and callous by the constant contemplation of what is vicious. The same sense of hopelessness, coupled with the feeling that public matters are not our concern, which leads us to put up with the slush and smoke and griminess of London, is, perhaps, at the root of the strange fact that people of the strictest morality are able to walk through the streets of London not only seeing but actually rubbing shoulders with its poverty, its misery, and its vice without a shudder; regarding it all, as we must suppose they do, as something entirely outside their lives and their responsibilities. This callousness is deep seated, and it will be as much as the united churches can accomplish to rouse up that public opinion which we are told is to be so all-powerful for good. The Bishop of London says that the operations of the new society will probably bring to light many things which are now carefully hidden away, and he hopes that when these things are brought into this full light they will perish. But with the example of the streets before us we can hardly be so hopeful. From the medical point of view it is the condition of the streets that should at once be dealt with, for this is the great and prominent cause of disease among those who are not wilfully vicious. The latter may be put on one side in regard to this question, but there are hundreds, nay thousands, of young men who would pass through their lives without coming to harm, and would marry without giving foul disease to their wives were the streets under more strict control. These men, who only slip under the influence of urgent temptation, get a slight attack of what is thought a slight disease, and when this gets well it passes out of mind. But when they marry and find their wives chronic invalids, or perhaps the victims of what is now known as "one child sterility"—able to become the mother of one child and no more—they curse their fate, or perhaps even curse their wives; they forget, however, to curse that municipal ineptitude which makes it impossible for a young fellow to attend a polytechnic lecture or a Bible-class without being exposed to solicitation.

The evils resulting from this condition of affairs have been much brought to the front since the discovery of the gonococcus, and since gynæcologists have recognised the very important role played by this micro-organism in the production of many of the most serious diseases special to women. Until comparatively recent years it was chiefly by its effect in disseminating syphilis that the "social evil" was regarded as a danger to the health of the general population, but the great development of gynæcology in recent years, together with the bacteriological investigations which have been made in regard to the origin of the diseases from which women suffer, has shown how frequently the gonococcus is at the root of the mischief. Without going so far as Noeggerath, who states that 80 per cent. of women are affected with latent gonorrhœa, it seems quite certain that a very considerable number of reputable women are so affected, and unless we are to entirely reject the efficacy of the microbe as a cause of disease, we must admit that one of the commonest causes of endometritis, of tubal disease, and of inflammation around the ovaries and uterus, with all their disastrous consequences, is a gradual upward spread of gonorrhœal infection. Speaking at the recent conference at Brussels Professor Neisser, of Breslau, to whose investigations we are chiefly indebted for our knowledge of the gonococcus, said that a large number of serious affections, especially in women, some of which are followed by irremediable sequelæ, diseases, the origin of which was formerly entirely unknown, are now recognised to be the results of infection by this micro-organism, and he spoke of the wide-spread prevalence of this infection as a "danger which threatens the human race." Perhaps this may, in a sense, be an exaggeration, but, in any case, modern knowledge tends greatly to widen our conception of the evils produced by venereal diseases, and especially by the one in question, and when we recognise how disastrous is their influence, not only on the habitually immoral, whom we may leave to wallow in their own vices, but upon the wives and even the children of those who without being immoral have merely been weak in their resistance to temptation, we cannot but feel that even beyond the duty which the Bishop of London urges of bringing into light the hidden moral evils which infest this London of ours, there is the still more urgent duty of putting a stop, in far greater degree than is now done, to the "open market" which at present is an offence to all decent people, and a constant danger to the health as well as to the morals of the people.

